# Resilience indicator traits in chickens: a systematic review

**DOI:** 10.1016/j.psj.2026.107258

**Published:** 2026-06-10

**Authors:** Lisa Büttgen, Malou van der Sluis, Stina Auerbach, Anne Collin, Abel B. Ekiri, Klaus Wimmers, Tiago Fernandes Farias, Hannes Bergmann, Esther D. Ellen

**Affiliations:** a Animal Breeding and Genomics, Wageningen University & Research, 6700 AH Wageningen, the Netherlands; b Institute of Epidemiology, Friedrich-Loeffler-Institut, Federal Research Institute for Animal Health, 17493 Greifswald - Riems Island, Germany; c INRAE, Université de Tours, BOA, 37380 Nouzilly, France; d Department of Comparative Biomedical Sciences, School of Veterinary Medicine, University of Surrey, GU2 7AL Guildford, United Kingdom; e Genetics and Genomics, Research Institute for Farm Animal Biology (FBN), 18196 Dummerstorf, Germany; f Faculty of Agriculture, Civil and Environmental Engineering, University Rostock, 18059 Rostock, Germany

**Keywords:** Resilience, Chicken, Systematic review, Traits, Breeding

## Abstract

The resilience of an animal is its ability to cope with short-term disturbances, including those caused by pathogens, through response and rapid recovery to its original state. Here, a systematic literature review was conducted to identify resilience indicators that have already been studied and implemented for chickens, along with the contexts or specific stressor under which they were examined. The literature review was based on predefined search criteria, including ‘chickens’ as the population of interest, a stress exposure description and the term ‘resilience’. Screening of titles and abstracts was assisted by the AI-based tool ASReview, followed by full text screening and analysis. According to selection criteria, we finally identified 33 relevant publications on resilience indicator traits used in chickens. Two of these studies analyzed chicken resilience based on routinely collected big data, while the remaining majority were small or medium scale studies and trials. The majority of the studies focused on immune (n = 13) or thermal challenges (either before or after hatching; n = 14), especially heat stress. A variety of resilience indicators were studied; most studies investigated production- or performance-related parameters and/or immunity or disease-related parameters. About half of the studies included genetics- or gene expression-related indicators. Investigation of behavioral indicators of resilience - other than feed intake - was limited (n = 4). Overall, this systematic review provides a comprehensive overview of published resilience indicators in chickens and highlights gaps for future research. This review also revealed a need for the controlled use of terms like resilience, robustness, resistance, tolerance or adaptability, and the relevance of assessing the phase of recovery after a short-term disturbance in the context of resilience.

## Introduction

Breeding objectives for poultry are evolving from a predominant focus on production traits to balanced breeding including more or novel traits such as robustness and other health and welfare traits (e.g., viruses; Neeteson-van Nieuwenhoven, et al. (2013). Breeding for increased resilience, which aligns well with the goals of balanced breeding (Berghof, et al., 2018), is an upcoming topic (Knap and Doeschl-Wilson, 2020). Here we define resilience as the ability of animals to cope with short-term disruptions, including those caused by pathogens, through response and rapid recovery to their original state. In this definition, the term ‘rapid’ is relative, as the (reasonably expectable) speed of recovery may differ between different types or magnitudes of stressors, and serves to compare recovery speeds between individuals, in which a faster recovery is considered an indicator of better resilience. It is closely linked to – but not the same as – resistance of an animal, which we here define as its capability to avoid or mitigate the negative effects that any type of challenge might cause to it. Another term that needs to be distinguished from resilience is robustness, as the robustness of an animal in production is the ability to maintain its functionality under a wide range of conditions and stressors without substantial change. Short-term disruptions can be caused by a wide range of stressors. For chickens, including broilers, layers and dual-purpose breeds, typical stressors and challenges in production systems include climate-related stressors (e.g., high or low temperatures; Gil, et al., 2023), biotic stressors (e.g., viruses; Botchway, et al., 2022), shortages in resources (e.g., nutrition or water; Gil, et al., 2023; Jiang, et al., 2016) or negative interactions with conspecifics (regrouping; Dabbou, et al., 2022).

Research has shown that breeding for resilience can have economic benefits in livestock production, through a reduction in the (labor and health) costs associated with controlling disease (e.g., Berghof, et al., 2018; Knap and Doeschl-Wilson, 2020). Furthermore, studies have shown favorable associations between resilience and health, longevity or fertility parameters (e.g., Poppe, et al., 2020 for cattle). Moreover, improved resilience can positively impact the welfare of animals. Increased resilience can result in a reduction in negative impacts of disease, and resilience is considered to be an aspect of positive welfare (Rault, et al., 2025). Even though the resilience of an individual is determined by the response to a short-term disturbance, resilience is connected on a long-term view to survival in the sense that under the same circumstances, resilient animals are expected to live longer and have a higher probability of survival than less resilient animals (Friggens, et al., 2022).

The implementation of resilience traits in breeding programs is still limited (Berghof, et al., 2018), and this might be due to a lack of suitable and effective tools for monitoring or estimating resilience traits in livestock (Knap and Doeschl-Wilson, 2020). This is even more challenging for poultry, as birds are often difficult to monitor at the individual level in large groups (Berghof, et al., 2018). Nonetheless, with the ongoing development of new techniques for large-scale data collection, such as computer-vision systems (Okinda, et al., 2020), there is potential for the development of novel phenotypes that can be recorded automatically and used as resilience indicators in poultry breeding programs (Berghof, et al., 2018).

Advances in genomic selection and management practices - alongside a deeper understanding of the genetic, physiological, and behavioral capacities of animals, as well as their defense mechanisms against biotic and abiotic stressors and their interactions with the environment - now enable the selection of more efficient and resilient chickens suited to diverse farming systems. Therefore, it is important to develop the essential knowledge and tools, to incorporate resilience traits into selective breeding and to establish comprehensive animal health management systems and interventions. To generate evidence to support future inclusion of resilience in chicken breeding programs, we identified four gaps and steps to be taken: 1) identify resilience indicators and their relationships with health, welfare, and production traits in chickens, 2) understand the effects and interactions of genetics, nutrition, and management practices on resilience and health traits in chickens, 3) create new models to enhance resilience and health at the population level, and 4) evaluate the potential of these new tools in chicken breeding programs.

As a first step, we hereby performed a systematic literature review of potential resilience indicator traits in chickens. A systematic review approach is often used to capture as many relevant studies as possible, to systematically select relevant records for review and to then describe the identified information. Many of the systematic reviews follow the PRISMA (preferred reporting items for systematic reviews and meta-analyses; Page, et al., 2021) and PRISMA-P (preferred reporting items for systematic review and meta-analysis protocols) guidelines (EFSA, 2010; Shamseer, et al., 2015). Recently, several tools based on the use of machine learning were introduced to support literature searches and screening (Burgard and Bittermann, 2023). However, the source code is openly available for only a few tools using active learning based on the user’s manual decision, to give the user an inclusion probability of unreviewed references (Burgard and Bittermann, 2023), such as ASReview (van de Schoot, et al., 2021) or FASTREAD, which in principle makes their actions verifiable and therefore scientifically reliable. The AI tool ASReview was found to restrict the effort for screening of titles and abstracts to manually screening only about 25% of the publications (van Dijk, et al., 2023) by re-ranking the remaining publications after each decision on the relevance of a publication to give the publication with the highest probability to be relevant next (van de Schoot, et al., 2021). Compared to conventional screening using the web application Rayyan (Ouzzani, et al., 2016) for semi-automated abstract and title screening, using the semi-automated tool ASReview is significantly less time-consuming, but nevertheless with a sufficient accuracy (Oami, et al., 2024). Therefore, we decided to use ASReview for the title and abstract screening in this systematic review.

Overall, this systematic literature review aimed to 1) identify and list resilience indicator traits which have been already applied in chickens to quantify or rank the birds’ resilience, 2) identify data processing methods which might be applicable for other production traits or other easily measurable phenotypic data in chickens or even other species, and 3) develop hypotheses for the development of more practically applicable or more precise resilience indicator traits in chickens.

## Materials and methods

### Identification and systematic review approach

The *a priori* protocol of this systematic review with a focus on sensitivity to identify most relevant records was pre-established and time stamped on the 24^th^ of April 2025. It was then shared among the partners of the European Partnership on Animal Health and Welfare in the working group “Reinforcement of animal resilience” for critical revision. A revised version was time-stamped on 21^st^ of May 2025 (see Supplementary data 1) before executing the literature search according to this *a priori* protocol. The protocol and review planning were conducted according to PRISMA guidelines (Shamseer, et al., 2015) as applicable to the research objective, broken down into four key components according to the PECO acronym (Population, Exposure, Comparator, Outcome; Morgan, et al., 2018): What resilience measures have been identified in chickens?

The systematic review approach entailed four main steps: identification of literature; screening of identified literature; data extraction, and; quality assessment. The process was designed to capture as many relevant studies as possible, systematically select relevant records for review, and then to describe the identified information on currently known resilience indicators in chickens. No meta-analyses were performed.

### Information sources and search strategy

Bibliographic searches were conducted in the following databases and search engines, using the full available time range of the respective databases: **PubMed** (MEDLINE) with literature from about 1946 to 2025, **Web of Science** with literature from about 1945 to 2025, and **Scopus** with literature from about 1970 to 2025. The following components were identified: *Population*: Domestic chickens (*Gallus gallus domesticus*), including chicks, hens and roosters. *Exposure*: Various stress conditions (heat stress, pathogen exposure, social stress, etc.)*. Comparator*: Groups of chickens not exposed to stress conditions or baseline measurements (not considered in the title and abstract screening, see further on)*. Outcome*: Measures of resilience (see Table 1).

**Table 1:** Review components and related keywords.

Chicken	Stress Exposure	Outcome
Broiler*	abiotic	resilien*
chicken*	aggressi*	
chicks	behavio*	
gallus	cannibalism	
hen*	challeng*	
poultry	climat*	
	confinement	
	disease	
	disturbance	
	endotoxin	
	environment*	
	exposure	
	feather pecking	
	housing	
	humidity	
	internal stimuli	
	nutrition	
	parasit*	
	pathogen	
	predators	
	resources	
	short term perturbations	
	short-term perturbations	
	social	
	stocking density	
	stress*	
	temperature	
	thermal	
	transport*	
	vaccination	
	infect*	

The search topics were reduced to the following three search terms: **Chicken** (Population) AND **Stress exposure** (Exposure) AND **Resilience** (Outcome). The comparator was not included in the search string to avoid narrowing the search and potentially excluding relevant studies, as was confirmed by a manual check of a subset of publications. The inclusion of a comparator in the search string could inadvertently filter out important articles where the comparator group is not explicitly mentioned or described in the same terms in the title or abstract - while being present according to the full text -, thus missing valuable data on resilience measures in chickens. The focus on population (chicken), exposure (stressor), and outcome (resilience) allowed the search to remain broad and to capture a wide range of studies.

Based on the identified search topics, relevant keywords and controlled vocabulary were identified to generate the final search string for database queries (see Table 1). In the search terms for the population, the term “layer*” was not included considering the double meaning of the word which can lead to a high number of non-chicken-related publications. A small-scale manual cross-check showed that nearly all relevant publications on layers also use in the abstract the term “hen”.

### Eligibility criteria

The eligibility criteria of reviewed studies directly flowed from the study objective and included the following:

• POPULATION & COMPARATOR: Describing livestock species of interest.
  Studies involving domestic chickens (*Gallus gallus domesticus*), including chicks, hens and roosters.
  Studies that included a comparison group of chickens, which could be 1) a group of chickens that was not exposed to the stress conditions, or 2) longitudinal measurements of periods with low stress exposure for chickens that experienced the stress conditions at another point in time.
• EXPOSURE: Describing the **stressors or challenges** the animals were exposed to.
  Studies investigating exposure of chicken to one or more stress conditions such as heat stress or pathogens.
• OUTCOME: Describing the animal performance.
  Studies that stated that they (intended to) measure resilience.
  Describing an **adaptive measure** or indicator (Test or method of interest): Studies that described specific adaptive measures or indicators used to assess resilience in chickens under stress conditions.
  Describing an **output** (e.g., resilience traits, proxy/indicator for resilience): Studies that described outputs such as resilience traits or indicators for resilience in chickens. This could include measures of how well chickens maintained their performance under stress, or indicators of adaptive capacity.

### Study records

*Data Management.* The literature searches were conducted on 21^st^ of May 2025. Searches were performed in the databases PubMed, Scopus, and Web of Science using the defined search strings. All retrieved search results were recorded in raw data files specific to each database. The recorded search results were merged into one file in ris-format through import into the reference management software EndNote (2020) for subsequent deduplication. The removal of duplicated records due to their retrieval from more than one database was done with ASySD version 25.1.1 (Hair, et al., 2023). The deduplicated records were imported into the AI-assisted tool ASReview (van de Schoot, et al., 2021) for re-ranking the records based on their relevance during title and abstract screening. De-duplicated titles and abstracts were also exported into a pre-defined digital spreadsheet (XLS format) to consolidate and organize the records during the review process.

***Selection Process****.* The literature selection process was tailored to address the main objectives of the systematic review by applying the following selection steps: calibration and refinement, initial screening, hierarchical review, screening process, and documentation.

*Calibration and refinement*. Before initiating the full screening process, a calibration using a small random subset of 25 records was conducted to set up the ASReview environment and to align reviewers’ decisions according to the *a priori* protocol. This helped to refine the selection criteria and ensure consistency in the application of the criteria. This subset, reviewed by both reviewers, was used to initialize the reordering of the publications by relevance by the ASReview tool.

*Hierarchical review*. The first reviewer (**R1**) reviewed the title and abstract of the publications in the order suggested by the AI-assisted tool ASReview (van de Schoot, et al., 2021), which is designed to re-rank the remaining publications after each decision on the relevance of a publication to give the publication with the highest probability to be relevant next. Records were assessed for their relevance based on the predefined criteria related to resilience measures in chickens (see *Screening process*). If R1 considered a record relevant, it was included for the further full-text screening. If R1 deemed a record irrelevant, the record was passed to the second reviewer (**R2**) for a second assessment up until the point where R1 had labelled 20 publications in a row as irrelevant. After 20 publications in a row were labelled as irrelevant, only R1 reviewed the remaining publications to check the accuracy of ASReview. R2 applied the same criteria as R1. If R2 considered the record relevant, it was included; otherwise, it was excluded. For records which were not excluded in the screening of titles and abstracts, the full text screening was conducted in the same manner. For full text screening, the selected publications were split among the authors.

*Screening process*. Reviewers followed a hierarchical set of selection questions and the publications were assessed against specific inclusion and exclusion criteria (see Supplementary data 1) until a record was determined to be ‘No’ for inclusion, or was kept as all inclusion criteria were met. Relevant records were included and irrelevant records were forwarded to a second reviewer for further assessment according to the procedure described above.

*Documentation.* Reasons for excluding records from the initial pool were recorded. This documentation ensured transparency and provided a basis for understanding the decision-making process throughout the review.

By adhering to these steps, we systematically evaluated records to identify and describe resilience measures in chickens exposed to stress, ensuring that only relevant studies were included for analysis. Exact questions used for the title and abstract screening are available in the *a priori* protocol in Supplementary data 1. All studies which were not excluded during the title and abstract screening or the full text screening were considered in the current study.

## Results and discussion

### Summary of reviewed studies

**Figure 1:**
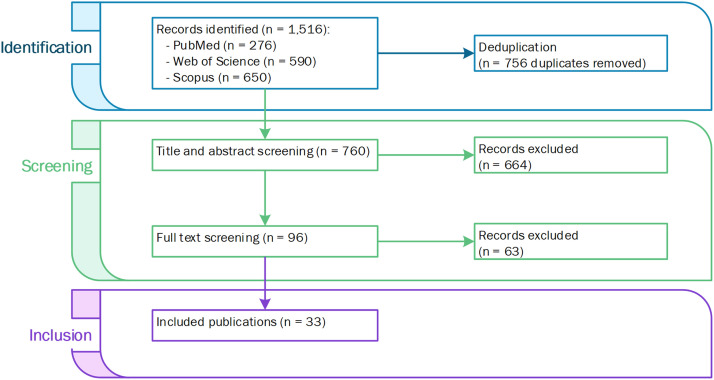
Flowchart of the identification, screening and inclusion of publications.

**Figure 2:**
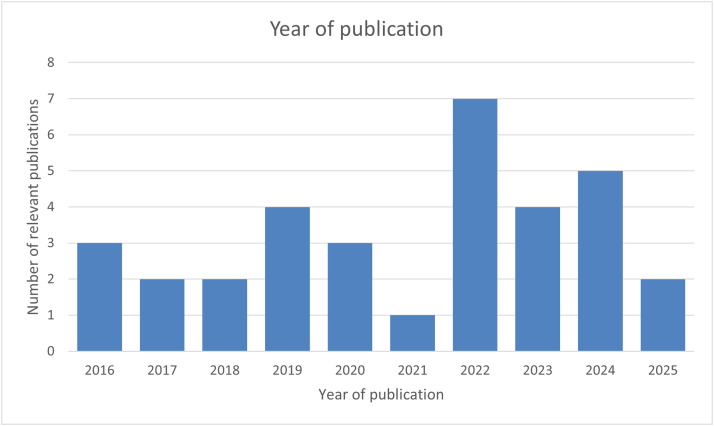
Distribution of the year of publication of the publications screened as relevant for resilience indicator traits in chickens.

Search string-based literature database queries resulted in 276 publications from PubMed, 590 publications from Web of Science and 650 publications from Scopus (Figure 1). After deduplication, in which 756 duplicates were removed, 760 publications remained for title and abstract screening. None of the 3 possible duplicate pairs flagged for the manual deduplication were considered a duplicate. After the title and abstract screening, 96 publications remained for full-text screening. Finally, after full text screening, 33 publications were scored as relevant for the current review on chicken resilience (Abdel-Fattah, et al., 2024; Banos, et al., 2025; Bedere, et al., 2022; Berghof, et al., 2024; Berghof, et al., 2019; Botchway, et al., 2022; Boulton, et al., 2018; Cramer, et al., 2019a; Cramer, et al., 2019b; Dabbou, et al., 2022; David, et al., 2019; Doekes, et al., 2023; Gangnat, et al., 2020; Gil, et al., 2023; Hundam, et al., 2025; Jiang, et al., 2016; Keerqin, et al., 2017; Kisliouk, et al., 2017; Koslova, et al., 2020; Loyau, et al., 2016; Mellouk, et al., 2024; Molenaar, et al., 2023; Naraballobh, et al., 2016; Nawaz, et al., 2024a; Nazar, et al., 2022; Park, et al., 2022; Putyora, et al., 2023; Rodrigues, et al., 2018; Ross, et al., 2020; Santos, et al., 2022; Tomczyk, et al., 2024; Wijnen, et al., 2022; Wijnen, et al., 2021). Most publications that were screened as a full text, but were ultimately excluded, did not describe resilience measures in chickens (52 out of 62 publications) based on the definition given in the introduction. The most frequent reason was that the studies’ operationalization of resilience did not explicitly capture the short-term recovery phase after a stressor, which was a required component of the more stringent definition of resilience applied in this review. The relevant studies were published between 2016 and 2025, with a maximum count of seven relevant publications in the year 2022 (Figure 2). With 18 publications (55% of the included publications) studying broilers, broilers were the most studied type of chicken, followed by laying hens studied in ten publications and dual-purpose breeds studied in three of the relevant studies. In eleven studies only females were studied and in ten only males were studied, while seven studies examined both sexes. For the remaining four studies, it was not specified. Two of the studies analyzed big data (both >65,000 birds), while the remaining studies were small or medium scale studies (<2,500 studied birds). In 18 of the relevant studies, there was a comparator group consisting of a group of birds not exposed to the stressor, in three studies the control group was exposed to less stress and in 16 studies there was a comparison by a time series of measures or longitudinal data.

**Figure 3:**
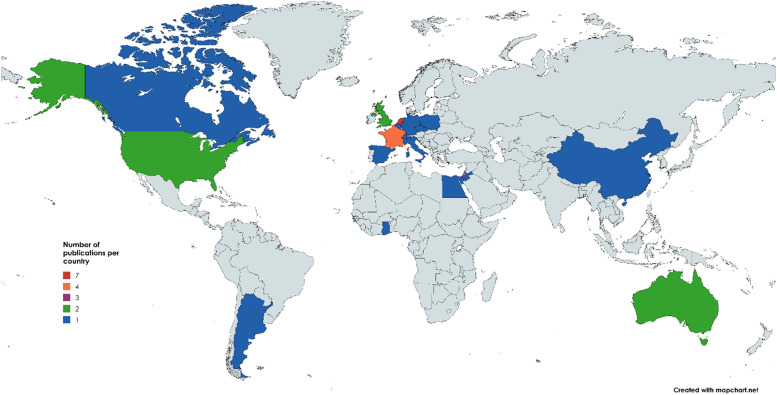
Number of publications of the publications screened as relevant for resilience indicator traits in chickens per country.

The relevant publications on chicken resilience were authored by persons working at research institutions and universities from five different continents (Figure 3). Nevertheless, the majority of studies (61%) had a first author affiliated with an institution located on the European continent, mainly from the Netherlands (7 publications) and France (4 publications).

### Type of stressor

When assessing the type of stressor examined in the studies, it appeared that the majority of studies focused on some form of immune challenge (39.4%) or a thermal challenge after hatching (24.2%; Table 2). For thermal stress, most studies focused on heat stress, although some also studied responses to cold stress.

**Table 2:** Overview of the reported stressors in the studies^1^.

Stressor category	Specific stressors
Immune challenge (n = 13)	Necrotic enteritis (n = 3), infectious bronchitis (n = 3), Eimeria (n = 3), Newcastle Disease (n = 2), E. coli (n = 1), avian leukosis (n = 1), lipopolysaccharide (n = 1)
Thermal stress - after hatching (n = 8)	Heat stress (n = 7), cold stress (n = 1), weather fluctuation (n = 1)
Thermal stress - before hatching (n = 6)	Embryonic heat conditioning (n = 3) embryonic heat stress (n = 2), embryonic cold stress (n = 2)
Nutritional stress (n = 5)	Omission of synthetic methionine (n = 1), reduced dietary metabolizable energy content (n = 1), fasting (n = 1), water restriction (n = 1), early versus delayed feeding after hatching (n = 2)
Other (n = 5)	Regrouping (n = 1), stress reactivity tests (n = 2), repeated opportunity test (n = 1), sleep disturbance (n = 1), different hatching systems (n = 1)
Longitudinal data without specific stressor (n = 4)	No specific stressor (n = 4)

1 some studies included multiple challenges, so the total numbers add up to more than the number of studies.

### Identified resilience indicator traits

Broadly looking at the types of resilience indicators, it appeared that the majority of the studies (64%) examined at least some production- or performance-related parameter, such as body weight (e.g., Berghof, et al., 2019), feed conversion ratio (Keerqin, et al., 2017) or egg production (Berghof, et al., 2024). A total of 19 out of 33 studies (58%) examined immunity or disease-related parameters, such as heterophil-to-lymphocyte ratios (e.g., Gil, et al., 2023), intestinal lesion scores (e.g., Boulton, et al., 2018) or antibody levels (e.g., Mellouk, et al., 2024). About half of the studies (48%) included a genetics- or gene expression-related indicator, such as epigenetic regulation of mRNA expression (e.g., Kisliouk, et al., 2017), gene expression in specific tissues (e.g., Naraballobh, et al., 2016) or genetic correlations between resilience-related traits (e.g., Berghof, et al., 2019). Of the 33 studies, 4 (12%) included one or more behavioral indicators other than feed intake. Feed intake – which could be considered behavior – was not included as a behavioral indicator here, as most studies that recorded feed intake appeared to do so with the aim of assessing feed conversion ratios or other growth-related aspects. The behavioral indicators reported in the four publications included fearfulness measurements such as tonic immobility (Dabbou, et al., 2022) or startle responses (Ross, et al., 2020), sleep behavior (Putyora, et al., 2023), or exploration and learning behavior (Nazar, et al., 2022). A complete overview of all indicator traits examined in each of the studies can be found in Supplementary data 2.

### Identified phenotype data processing methods

Most studies analyzed chicken resilience using classical hypothesis-testing approaches with fixed-effects models (e.g., t-tests, ANOVA, and general linear models) to compare mean responses between groups, whereas others used mixed-effects models (linear or generalized) to account for random factors and repeated measures (Supplementary data 2 Table S2.1). In addition, some studies used genetic and heritability models or survival analysis. A smaller subset quantified resilience using longitudinal, variability-based metrics that capture fluctuations over time rather than between-group differences. For the analysis of longitudinal data, five studies used the natural logarithm of the variance (LNvar), skewness, and autocorrelation. Four of the five studies did not study a specific stressor, while the study by Wijnen, et al. (2022) applied LNvar, skewness, and lag-one autocorrelation to measure resilience to colibacillosis.

### Measuring resilience: definitions and challenges

During this study, it became apparent that measuring resilience is not a trivial matter. The topic of recovery is an important part of the resilience definition used in the current study (i.e., the ability of animals to cope with short-term disruptions, including those caused by pathogens, through response and rapid recovery to its original state), but it was not always clearly addressed in the reviewed studies. According to a survey by Słomian, et al. (2025), most scientists studying animal resilience agree with the here-used definition of resilience (>80%). However, in general, during the screening of potentially relevant publications, it was noticeable that very few authors clearly defined resilience in the introduction (or elsewhere in the paper), even when resilience appeared to be the thematic focus of the study.

In the 33 studies that were screened as relevant for chicken resilience, only five publications appeared to provide a clear definition of resilience (Berghof, et al., 2024; Berghof, et al., 2019; Rodrigues, et al., 2018; Ross, et al., 2020; Wijnen, et al., 2021). While the definition of Berghof, et al. (2019); Rodrigues, et al. (2018) and Berghof, et al. (2024) also included recovery or rapid return to the state before the disturbance, in the publications of Ross, et al. (2020) and Wijnen, et al. (2021), resilience was described more by a low impact of a stressor or disturbance, but also not stated as a formal definition. To ascertain that the aspect of recovery was present in the studies included in this review (as required by our definition), we clarified a decision rule during the full text screening to help us implement this requirement in a consistent manner, in which at least two measurements were needed to capture recovery, of which at least one measurement should be taken before or during the disruption and at least one measurement should be taken after the stressor occurred. Accordingly, longitudinal data including measurements after the disruption were considered sufficient. In case there was no measurement before or during the disruption, the criterion could alternatively be met by including a control group not experiencing a stressor or disruption. Such a control group - instead of measurements on the same individuals - was included to account for resilience measures which are invasive or require slaughtering the birds (such as gene expression analysis in different organs, as was done by Nawaz, et al., 2024b).

The type of the resilience measure also depended on the type of objective for studying resilience. It was observed that if the aim of the study was exploring the biology, effects and mechanisms of animal resilience, there were often fewer but more specific measurements made, such as blood samples (e.g., Gil, et al., 2023) and traits which could only be obtained after slaughtering animals. When the aim of the study was to define a trait, which could be used to select and breed more resilient animals (e.g., David, et al., 2019), then these kinds of traits were less relevant or less precise, but routinely measurable on a large scale of animals (e.g., Berghof, et al., 2019).

Overall, measuring resilience including the phase of recovery after a perturbation seems to be even more challenging in chickens than in larger animal species such as cattle, since the amount, frequency and nature of routinely collected data differ. Due to the relatively short lifetime of broilers, a high frequency of measurements is necessary in order to be able to study longitudinal patterns in routinely collected data, but these are often not available. For example, weight measurements in chickens are - in the common production systems - too infrequently recorded to cover the effect of short-term disturbances. In larger animals, body weight can be measured more often, at the individual level, and in an automated way (e.g., Gorssen, et al., 2023 used longitudinal data of weight, feed intake and feeding behavior for variance-based resilience measures in pigs), while body weight in chickens is generally measured manually, e.g., approximately every four weeks (Berghof, et al., 2019). Another challenge lies in the nature of the data that can be collected. For example, while the laying rate in laying hens can be recorded daily, it is a discrete measure and therefore - in comparison to, for example, daily milk yield of cows (used by Poppe, et al., 2020 to model resilience indicator traits) - not easy to use to model recovery on a hen-individual level. Additionally, in chickens it is less common to have longitudinal behavioral data, which could be used to build a resilience indicator trait, than in larger animals. For example, step counts can be used in cattle (Poppe, et al., 2022), while for chickens, sensors are often too large or heavy to be implemented at all ages (Ellen, et al., 2019). Techniques such as computer vision (Okinda, et al., 2020) may be necessary to overcome this issue but are challenging due to all chickens looking highly similar, which complicates individual recognition.

### Coverage of different types of chicken, regions of the world, types of stressors and types of resilience indicators

The majority of the included studies focused on broilers. Especially for heat stress-related studies, this focus on broilers appears logical: due to their higher body weight, broilers might be more affected by high temperatures (Gogoi, et al., 2021) compared to laying hens or dual-purpose breeds, making this more relevant to study in broilers. Studying resilience in broilers may, however, be even more challenging than in laying hens or dual-purpose chickens, for two reasons. First, the short commercial life span of broiler chickens hampers data collection across long periods of time, and therefore, “long-term” recovery is difficult to assess. Second, the fast decrease in activity that is observed in broilers as they age (e.g., Gogoi, et al., 2021) makes it challenging to record behavioral indicators at later ages: the broilers might be very inactive regardless of a challenge being present (see e.g., Duenk, et al., 2024). Nonetheless, broilers appear to be the most-studied type of chickens in terms of resilience. However, as broiler chickens may differ considerably from laying hens and dual-purpose chickens (e.g., in terms of growth rates and body weights (Cooke, et al., 2003), cecal microbial composition (Willson, et al., 2022), and responses to vaccines (Groves, et al., 2015), it would be advisable to study resilience in these other types of chickens in more detail too. Studying resilience in dual-purpose or layer breeds has the added benefit that resilience may be easier and more insightful to study, given the longer commercial life allowing for assessment of short-term stressors that have longer recovery periods. Moreover, repeated measurements on the same individuals over longer periods of time would provide more detailed insight into recovery patterns, and would allow examination of different stressors at different points in individuals’ lives. This can contribute to assessing sensitive periods or resilience across different types of stressors for the same individuals (i.e., correlated responses across different stressors).

Regarding the type of stressors studied, it became clear that the majority of included studies focused on immune challenges or thermal stress. These are challenges that appear to be common in chickens’ lives (although the exact climate challenges depend on the region) and are therefore highly relevant to study. However, there are other potential stressors that chickens might come across in life, such as failures in feed and water supply, lighting or other system failures inside the house, stressful social interactions (e.g., feather pecking), or handling by humans (e.g., for health checks or transport). More attention to these other types of stressors in future research could contribute to a more complete understanding of resilience in chickens.

In the assessed studies, many different indicators of resilience were reported. However, broadly speaking, behavioral indicators (other than feed intake) appeared to be sparse (present in only 12% of the studies assessed here). This suggests a currently limited insight into short-term responses, as behavioral responses might be the first indicator of discomfort in chickens (Duenk, et al., 2024), changing much faster than body weight or egg production, for example.

### Reflection on the methodological approach of the systematic review

Designing a search string for a systematic review is challenging and requires a balance between a complete list of search terms and exclusion of search terms which have more than the intended meaning or a very broad meaning. In the current systematic review, the terms ‘layer’ and ‘laying’ were not included in the search string, as these are also broadly used terms in the context of diverse topics and the effort for manual screening of all abstracts found by such a search string would have been prohibitively high. For these terms, it was observed that the term ‘hen’ (which was included in the search string) was generally mentioned in the abstract as well, resulting in inclusion of the publication in the initial data set. Nevertheless, it cannot be ruled out that the higher number of broiler publications identified is partly attributable to this choice of search terms.

One way to overcome this issue, as well as in general high numbers of publications found by a search string, is the use of AI to speed up at minimum the title and abstract screening. One option for such an AI tool is ASReview (van de Schoot, et al., 2021), which sorts the remaining abstracts after each manual decision whether an abstract might be relevant or not, based on the probability of the remaining abstracts being relevant. In the current systematic review, ASReview was used to screen all titles and abstracts by the first reviewer, who forwarded the abstracts screened as irrelevant to a second reviewer up to the stop criterion of 20 irrelevant publications in a row (as described in the methods section). Afterwards, the remaining abstracts were only screened by the first reviewer for cross-validation of the method in the field of animal resilience. After a stop criterion of 50 irrelevant publications in a row, as considered, e.g., by Boetje and van de Schoot (2024), the first reviewer screened eleven further abstracts as possibly relevant, but none of these were considered relevant after conducting the full text screening. Since the final assessment is relevant for a systematic review, we consider the use of ASReview to be valuable and recommend this tool for further reviews in similar subject areas. Nevertheless, we see potential improvement regarding the way we applied ASReview. ASReview was not pretrained by known targeted publications but by the decision on 25 random publications. It might be beneficial to either do already the full text screening for the potentially relevant abstracts before screening one as relevant or entering relevant publications based on prior knowledge. For the pretraining with irrelevant abstracts, we do not see the need for methodological improvement over just screening random abstracts.

### Suggestions for improvement or development of resilience indicator traits

Based on this systematic review, several recommendations can be made for future research:

First, when reporting results of an experiment, it is helpful to clearly distinguish and define whether resilience or resistance, tolerance, robustness, or adaptability or a combination of those are studied. Based on our experience from this review, we recommend that authors clearly define these terms at the outset and, if necessary, distinguish between them.

Second, it is important to clarify - if necessary - whether some aspect(s) of resilience could not precisely be captured with the experiment or data analysis, and to describe the underlying reasons and how this was addressed in the study.

Third, when studying animal resilience, it is important to pay attention to finding a way to also capture the animals’ recovery after the disturbance, as this is an important component of resilience (in our view and definition). This can be realized most straightforwardly by either analyzing time series data available at a sufficient frequency or by taking at least one sample for each individual before and after the disturbance - and ideally also during the disturbance if it is unclear if every individual is impacted to the same extent by the disturbance.

Fourth, it is important to consider the time frame of the occurrence of the stressor or disturbance, since resilience is at its core defined as reaction to a short-term disturbance. For this reason, any long-term - or even continuous - exposure to stressors, potentially resulting in chronic stress in the animal, are not considered suitable for studying resilience. It is difficult to set a fixed criterion for what can be considered short-term. Most importantly, a temporary stressor should be followed by a study period that is sufficiently long to allow for complete recovery. To give an example, heat stress for a matter of hours or days could be considered as a short-term stressor that broilers can recover from, whereas weeks of heat stress would perhaps not allow for a full recovery period afterwards, due to the limited commercial life of broilers.

Fifth, for future research, it would be advised to additionally study stressors other than immune or temperature challenges (for which quite some knowledge is already available). It would furthermore be insightful to study behavior as a potential resilience indicator in more detail: behavioral responses might be the first indicator of discomfort in chickens, and one of the first observable manners in which chickens adapt to a challenging situation.

Overall, this systematic review provides the first foundational and structured synthesis of the existing scientific evidence on resilience indicators in chickens. It highlights that animal resilience is an important and emerging topic of research. Even though some gaps were identified here – both in terms of stressors studied and types of resilience indicators used – important first insights into chicken resilience are already available in the scientific literature. These insights can contribute to create more sustainable chicken production systems in the future.

## Data availability statement

The original contributions presented in the study are included in the article/Supplementary Material. Further inquiries can be directed to the corresponding author.

## CRediT authorship contribution statement

**Lisa Büttgen:** Writing – review & editing, Writing – original draft, Visualization, Validation, Project administration, Methodology, Investigation, Formal analysis, Data curation, Conceptualization. **Malou van der Sluis:** Writing – review & editing, Writing – original draft, Visualization, Validation, Methodology, Investigation, Formal analysis, Data curation, Conceptualization. **Stina Auerbach:** Writing – review & editing, Methodology, Conceptualization. **Anne Collin:** Writing – review & editing, Investigation, Conceptualization. **Abel B. Ekiri:** Writing – review & editing, Investigation. **Klaus Wimmers:** Writing – review & editing, Investigation. **Tiago Fernandes Farias:** Writing – review & editing, Investigation. **Hannes Bergmann:** Writing – review & editing, Methodology, Conceptualization. **Esther D. Ellen:** Writing – review & editing, Supervision, Project administration, Methodology, Investigation, Funding acquisition, Conceptualization.

## Disclosures

The authors declare that they have no known competing financial interests or personal relationships that could have appeared to influence the work reported in this paper.

## Appendix Supplementary materials

Supplementary Material Supplementary data 1: Overview of resilience indicators studied in the 33 relevant publications Image, application 1

## Footnotes

Supplementary material associated with this article can be found, in the online version, at doi:10.1016/j.psj.2026.107258.
