# Supplementary material for: Resilience indicator traits in chickens: a systematic review
Source: Poult Sci. 2026 Jun 10;105(9):107258. doi: 10.1016/j.psj.2026.107258 (PMC13311826; doi:10.1016/j.psj.2026.107258)
Supplement: Supplementary file 1 — Supplementary Material Supplementary data 1: Overview of resilience indicators studied in the 33 relevant publications [file mmc1.docx]

**Supplementary data 1**

**Table S1** shows the resilience indicators and stressors that were studied in the 33 publications that were considered relevant for assessing chicken resilience.

**Table S1. Overview of resilience indicators studied in the 33 relevant publications.**

| **Publication^1^** | **Stressor** | **Resilience indicator(s)** | **Data analysis method** |
| --- | --- | --- | --- |
| Abdel-Fattah et al. (2024) | Embryonic heat conditioning; SPIDES; heat stress | Egg quality (egg weight, egg shape index, yolk weight, yolk index, albumen weight, albumen index, albumen pH, albumen height) and egg weight loss; body weight; body weight gain; feed consumption; FCR; carcass weight; relative weights of breast muscles (pectoralis major and pectoralis minor) and some internal organs (liver, heart); blood parameters (total protein concentration, total albumin concentration, globulin levels, cholesterol, high-density lipoprotein, triglycerides, enzymatic activity of AST and ALT); plasma hormones (triiodothyronine level); histology and cytology of skeletal muscle (myocyte count, myocyte diameters). | GLM |
| Banos et al. (2025) | Weather fluctuation | Body weight; average daily gain; calculated individual reaction norms in BW as resilience phenotype | Regression model; random regression model; calculated individual reaction norms in BW (calculated by adding the individual deviations to the corresponding population norm); mixed model; linear mixed model |
| Bedere et al. (2022) | Longitudinal | (Deviations in) egg production; heritability and genetic correlations with egg production | LNvar; skewness; autocorrelation; bivariate analyses; animal mode; sire models; univariate models |
| Berghof et al. (2019) | Longitudinal | (Deviations in) body weight; genetics of these deviations and correlations with immunity and disease resistance | LNvar; skewness; autocorrelation; linear animal model; bivariate analyses; Cox proportional hazards model; general linear model |
| Berghof et al. (2024) | Longitudinal | (Deviations in) egg production; genetics and correlations with egg production and antibody traits | LNvar; skewness; autocorrelation; bivariate analysis; univariate analyses; linear animal model |
| Botchway et al. (2022) | Newcastle Disease virus challenge | Growth rates; viral load in tear samples; viral cleareance rate; antibody levels in blood; lesion scores in trachea, proventriculus, intestines and cecal tonsils, and in brain, lungs and spleen | Linear mixed models; univariate animal models (genetic analyses) |
| Boulton et al. (2018) | Eimeria challenge | Weight gain, caecal lesion score; intestinal inflammation (circulating interleukin-10); SNPs correlated with weight gain | Linear univariate model; GWAS |
| Cramer et al. (2019a) | Heat stress | Body temperature; DNA CpG methylation (5mC) and hydroxymethylation (5hmC) at the CRH intron in paraventricular nucleus; histone acetylation enzyme GCN5 | Two-tailed t-test; one-way ANOVA; two-way ANOVA |
| Cramer et al. (2019b) | Heat stress | Body temperature; DNA methylation and hydroxymethylation in paraventricular nucleus of hypothalamus and CHR intron | Two-tailed t-test; one-way ANOVA; two-way ANOVA |
| Dabbou et al. (2022) | Regrouping | Morbidity; mortality; egg production (daily egg-laying rate); tonic immobility; plumage condition; hematological parameters (total erythrocytes and leucocytes, heterophil:lymphocyte ratio; fecal corticosterone metabolites concentration | Chi-square test; GLM-repeated measures (mixed between-within subject models) |
| David et al. (2019) | Embryonic heat conditioning | Genome-wide distribution, in hypothalamus and muscle tissues, of two histone post-translational modifications, H3K4me3 and H3K27me3 | Generalized linear model; likelihood ratio tests; exact Fisher's tests; core analysis; Wilcoxon signed rank test |
| Doekes et al. (2023) | Longitudinal | (Deviations in) egg production; GWAS for this and natural antibodies | LNvar; skewness; autocorrelation; linear mixed models |
| Gangnat et al. (2020) | Nutritional challenges (effect of the omission of synthetic methionine; reduced dietary metabolisable content) | Egg production; egg weight; feed intake; FCR; body weight; egg quality (egg shape index, egg shell colour, shell strength, yolk weight, egg weight, egg white weight, Haugh unit); metabolisability parameters (energy and nitrogen metabolisability) | GLM; MIXED |
| Gil et al. (2023) | Cold stress; water restriction; heat stress | Heterophil to lymphocyte ratio | Mixed linear models; general linear models |
| Hundam et al. (2025) | Embryonic heat conditioning; LPS challenge | Body weight; body temperature; microbiome analysis of cecal content samples | Two-way ANOVA, one-way ANOVA |
| Jiang et al. (2015) | Fasting stress | Epigenetic modification and regulation of the gene for brain-derived neurotrophic factor (Bdnf) in paraventricular nucleus and forebrain | Two-way ANOVA; one-way ANOVA; GLM |
| Keerqin et al. (2017) | Necrotic enteritis challenge | Body weight; feed intake; FCR; intestinal lesion score; intestinal bacteria count | ANOVA |
| Kisliouk et al. (2017) | Heat stress | Epigenetic regulation of mRNA expression of the molecular chaperone heat-shock protein (HSP) 70 (HSPA2) in hypothalamus | Student's t-tests; one-way ANOVAs |
| Koslová et al. (2020) | Avian leukosis virus | Quantitative reverse transcriptase PCR to detect viral genomes from blood serum | Comparison of results |
| Loyau et al. (2016) | Heat stress | Body surface temperature (wing, comb and shank); egg quality (egg width, egg weight, yolk weight, shell weight, albumen weight, yolk proportion, shell proportion, albumen proportion, egg shape index, yolk colour, shell colour, shell breaking strength, shell mechanical stiffness, albumen height); feather quality | GLM; genetic/heritability analysis (multivariate analysis) |
| Mellouk et al. (2024) | Infectious bronchitis challenge | Average daily gain; total IgY and IBV-specific antibodies and leukocytes in blood; splenic leukocytes (IFN-γ spots; liver); cytokine expression levels/concentrations (liver) | ANOVA; independent two-tailed t-test; correlation analysis |
| Molenaar et al. (2023) | Newcastle Disease vaccination; infectious bronchitis vaccination; different hatching systems | Eggshell temperature before hatching; chick quality indicators (weight, length, navel condition, red hocks, red beaks); body weight; mortality; blood samples for antibodies; litter quality; footpad dermatitis; hock burn; cleanliness; skin lesions; gait score; inflammation and epithelial integrity related gene expression in the trachea; epithelial damage of trachea; inflammatory mucosal changes in trachea | MIXED; GLIMMIX |
| Naraballobh et al. (2016) | Embryonic temperature stress (low and high) | Gene expression in breast muscle | Mixed procedure in JMP Genomics |
| Nawaz et al. (2024) | Heat stress | Gene expression analysis (liver, hypothalamus, pectoral muscle); live weight; feed intake; protein efficiency ratio; weight gain ratio; body organ index; meat quality indicators (meat colour of pectoral and thigh muscle, muscle shear force, water loss rate, cooking loss, drip loss); histopathology (liver tissue) | General linear models; unpaired Student's t-tests |
| Nazar et al. (2022) | Tonic immobility test; repeated opportunity test | Immune status indicators (lymphoproliferative response to phytohaemagglutinin-p, interferon gamma plasmatic concentration, natural antibodies against sheep red blood cells, heterophil/lymphocyte ratio); tonic immobility for fearfulness (number of attempts to induce TI, latency to first head movement, latency to first vocalisation, and TI duration); repeated opportunity test for ability to adapt and take opportunities (latency to approach mid and then near part of pen, latency to peck bowl on ground, latency to jump up onto person, latency to peck bowl on person's lap, number of chicks in each area of pen, number of pecks at bowl, number of worms eaten from bowl) | Mixed models; linear mixed models; general linear model; lineal discriminant analysis |
| Park et al. (2022) | E. maxima challenge | Body weight/growth; gut (jejunal) lesion scores; fecal oocyst shedding; transcriptomic analysis (jejunal samples; cytokines, Aryl Hydrocarbon Receptors, tight junction proteins, nutrient transporters) | Proc GLM; PROC MIXED (mixed model) |
| Putyora et al. (2023) | Sleep disturbance | EEG data (slow wave sleep, rapid eye movement sleep; wakefulness); behaviour (awake; resting; sleep) | Linear mixed models |
| Rodrigues et al. (2018) | Necrotic enteritis challenge | Necrotic enteritis lesions in intestinal tissues (duodenum, jejunum, ileum); pH of crop, gizzard, proximal jejunum, proximal ileum and ceca; intestinal contents of distal jejunum and distal ileum for digestibility studies, proximal jejunum for viscosity assessment, and proximal ileum and ceca for short chain fatty acids; mortality; feed intake; weight gain; FCR | ANOVA, Mann-Whitney U test |
| Ross et al. (2020) | Stress reactivity tests | Startle reflex amplitude; comb temperature (i.e. measure of peak magnitude of autonomic stress response); latency for comb temperature to return to pretest level (i.e., duration of autonomic response) | Mixed effects GLM |
| Santos et al. (2022) | Eimeria challenge | Mortality; body weight (gain); feed intake; FCR; oocyst shedding in fecal samples; Eimeria lesion scores in duodenum, jejunum and caeca; cecal weight; litter quality; footpad lesions | ANOVA; linear mixed model; logistic regression model |
| Tomczyk et al. (2024) | Infectious bronchitis challenge | Antibody levels (in serum, for chicken anaemia virus (CAV), avian orthoreovirus (ARV), infectious bursal disease virus (IBD), infectious bronchitis virus (IBV) and fowl adenovirus (FAdV), and Mycoplasma gallisepticum and M. synoviae); presence of pathogens; body mass index; health parameters; mortality rate; anatomopathological and microbiological examinations (heart, liver, spleen, gizzard, intestines, lungs, and kidneys were collected and changes in them were described) | Mann-Whitney U test; student's t-test |
| Wijnen et al. (2021) | Necrotic enteritis challenge; embryonic temperature stress; early versus delayed feeding | Mortality; body weight gain; disease morbidity (gut lesions, coccidiosis, dysbacteriosis, shedding of oocysts, footpad dermatitis, and natural antibody levels in blood) | Fixed-effects model; mixed effects model; two-way ANOVA; PROC MIXED; PROC GLIMMIX |
| Wijnen et al. (2022) | E. coli challenge; embryonic temperature stress; early versus delayed feeding | Disease morbidity (lesions in thoracic air sac, pericardium and serosal surface of liver, lesion severity); local infection (E. coli in air sacs); systemic infection (E. coli in blood); body weight over time (LNvar, skewness and lag-one autocorrelation of BW deviations); mortality | Fixed-effects model; mixed effects model; two-way ANOVA; PROC MIXED; PROC GLIMMIX; survival analysis (PROC PHREG); LNvar, skewness, and lag-one autocorrelation of standardized BW deviations |

^1^ For full references, see full text of the main article.
ANOVA = Analysis of Variance; BW = Body Weight; GLIMMIX = Generalized Linear Mixed Models Procedure; GLM = General Linear Model; GWAS = Genome-Wide Association Study; LNvar = Natural Logarithm of Variance; MIXED = Mixed Model Procedure; PROC GLIMMIX = Procedure for Generalized Linear Mixed Models; PROC GLM = Procedure for General Linear Models; PROC MIXED = Procedure for Mixed Models; PROC PHREG = Procedure for Proportional Hazards Regression
